# Mechanical Atresia Vaginæ

**Published:** 1881-05

**Authors:** 


### MECHANICAL ATRESIA VAGINÆ.

About two years since I was consulted by a woman not far from twenty years of age, who had been married six months. She gave the following history of her case, which became more and more interesting to me as a continued treatment developed all its peculiarities.

Her husband was possessed of strong passions and wished for sexual commerce very frequently. Upon her part each sexual act caused the most intense suffering, and she had come to dread his approaches more and more until for the past few weeks the intolerable anguish which she suffered had led her mate to advise her to consult a physician.

A vaginal examination disclosed the following: The vagina was somewhat elongated, its diameter was less than three fourths of an inch, indeed I was unable to introduce my finger for more than an inch, and the slightest inward pressure caused my patient to cry out from pain the more acute. I discovered, by means of a small speculum, that a prominent ridge extended the entire length of the vagina upon its anterior surface. This ridge was hard and fixed. The whole vaginal tube seemed to be entirely undilatable, while the lining membrane was in a state of active inflammation, no doubt from forcible congress. I asked her history to her disease and she said as follows: "When I was about ten years old I went with other girls after berries. In our course we reached a stone fence upon the further side of which there was a fringe of elder stubs, about two feet high, with sharpened extremities. I was the last to get over, my foot slipped, and as I fell one of the sharp sticks entered my vagina, and I fainted from pain and fright; they carried me to the nearest house, called my mother, and one physician after another was called till three of the best in town were with me. They found a piece of splintered and knotty wood in my body and it was more than two hours before they could extract it. It was eight inches long and had passed up the vagina into the cavity of the abdomen between the womb and bladder, lacerating the vagina its whole length. I was confined to the house for over two years." The result of this terrible injury was a firm cicatrix upon the anterior wall of the vagina, fastening the tube to the bladder very rigidly. The patient wished to be cured if possible, and indeed I had learned before this stage of the examination that she was well advanced in pregnancy, so an operation was in the highest degree necessary. I called a neighboring physician to aid me in administering ether, and when she was anæsthetised I proceeded to operate, and with my knife blade and handle, etc., I at length succeeded in separating the cicatrix from the adjacent tissues, then dilating the tube I packed it carefully and firmly with lint well saturated with antiseptics. I continued this pluging till the wound was well healed and, that too, with no contraction of its normal diameter. I forbade any intercourse till a year after labor, supposing all went well up to that time. Soon after a favorable issue of this treatment I lost sight of my patient.

A year or so after this I received a letter from her in which I was informed that she was the mother of a healthy boy, that the labor was favorable, with no laceration, and then came many expressions of gratitude for her deliverance from so grave a complication.—*Boston Medical and Surgical Journal.*

